# Serum Glycated Albumin Levels Are Affected by Alcohol in Men of the Jinuo Ethnic Group in China

**DOI:** 10.1155/2021/6627074

**Published:** 2021-02-10

**Authors:** Chaoyu Zhu, Xuhong Hou, Ming Li, Qingyi Sun, Huijuan Lu, Yuqian Bao, Li Wei, Weiping Jia, Fusong Jiang

**Affiliations:** Department of Endocrinology and Metabolism, Shanghai Jiao Tong University Affiliated Sixth People's Hospital, Shanghai Diabetes Institute, Shanghai Clinical Center for Diabetes, Shanghai Key Clinical Center for Metabolic Disease, Shanghai, China

## Abstract

**Aim:**

To investigate the effects of alcohol on serum glycated albumin (GA) levels in Chinese men.

**Methods:**

A total of 2314 male subjects from the Jinuo ethnic group in China were enrolled. Of these, 986 subjects drank alcohol frequently and 404 subjects did not. Lifestyle information was gathered by using a questionnaire, and measurements of blood pressure, body mass index, blood glucose level, liver function, and kidney function were collected. GA was measured by using an enzymatic method. Frequent drinking was defined as a history of drinking ethanol > 80 g/d within the past two weeks. Nondrinking was defined as no alcohol consumption in the past three months. Subjects with an alcohol intake between 0 and 80 g/d in the past two weeks were included in the drinking-occasionally group. Analysis of variance (ANOVA), correlation analysis, and linear regression were used to evaluate the effects of drinking on serum GA levels. Decision tree regression (DTR) algorithm was used to evaluate the effect of features (variables) on GA levels.

**Results:**

We found that male subjects who drank frequently had significantly lower serum GA levels than subjects who did not drink (13.0 ± 1.7 vs. 14.1 ± 3.7, *p* < 0.05). Spearman's correlation analysis calculated a coefficient of −0.152 between drinking and GA (*p* < 0.005). Linear regression established that drinking was an independent predictor for GA levels with a standardized regression coefficient of −0.144 (*p* < 0.05). Decision tree regression showed that the effect of drinking on GA levels (0.0283) is five times higher than that of smoking (0.0057).

**Conclusions:**

Frequent alcohol consumption could result in decreased GA levels in men of the Jinuo ethnic group in China.

## 1. Introduction

Serum glycated albumin (GA) is a nonenzymatic glycation product of albumin and glucose [1]. Since the half-life of albumin is 17–19 days, GA can reflect the average levels of blood glucose within the previous 2–4 weeks and can compensate for a deficiency of glycosylated hemoglobin (HbA1c), which reflects the average blood glucose level of the previous 8–12 weeks [2, 3]. GA has been widely used in clinical practice to assess blood glucose levels of patients before and after treatment, especially in patients who are at an early stage [4, 5]. GA has also been associated with chronic complications of diabetes mellitus (DM) and can be used as a predictor of DM complications [6, 7].

Previous work has demonstrated that GA levels are influenced by various factors [8, 9]. Concentrations of HbA1c are affected by erythrocyte replacement velocity. Similarly, GA levels are affected by the rate of serum albumin renewal. Albumin metabolism is affected by many factors. Alcohol consumption affects the metabolism of blood glucose and liver function [10]. Additionally, it has been reported that the concentration of glycated albumin in nondiabetic men in Japan can be reduced by drinking or smoking [11, 12]. However, the interaction between smoking and drinking has not been fully explored and the role of these two factors in patients with diabetes should be considered in clinical practice. It is known that drinking or smoking are common behaviors among Chinese men [13]. This study aimed to investigate the effects of drinking and smoking on GA levels among men in the Chinese community, including those with diabetes, impaired glucose regulation (IGR), and normal glucose tolerance (NGT).

## 2. Materials and Methods

### 2.1. Definitions

DM and IGR were diagnosed based on the diagnostic WHO 1999 criteria using the results of their 75 g oral glucose tolerance test (OGTT) without medical treatment. Drinking-frequently was defined as a history of drinking, the equivalent > 80 g/d of ethanol within the past two weeks. Nondrinking was defined as no alcohol consumption in the past three months. If the alcohol intake was between 0 and 80 g/d in the past two weeks, the subject was classified as drinking-occasionally. Smoking frequently was defined as smoking at least 1 cigarette per day for the past three months. If a subject had not smoked in the past three months, he was classified as no-smoking. Those who smoked some cigarettes but less than 1 cigarette daily in the past three months were classified as smoking-occasionally. Fibrosis index based on the 4-factor (FIB-4) value was used to evaluate liver fibrosis, FIB-4 = age × AST/PLT × $\sqrt{\text{ALT}}$ [14].

### 2.2. Study Subjects

From January to May 2012, a cross-sectional survey was conducted among men aged 18–75 years old from the Jinuo ethnic group in Yunnan Province, China. The study protocol was followed in accordance with the Helsinki Declaration and was approved by the Shanghai Jiao Tong University Affiliated Sixth People's Hospital's ethics committee. Informed consent was obtained from all participants prior to the survey. All subjects underwent an oral glucose tolerance test (OGTT), GA measurement, serum liver function, and renal function test.

### 2.3. GA Measurement

Liquid enzymatic determination of GA (Lucica®GA-L kit, Asahi Kasei Corporation, Tokyo, Japan) was performed using an Olympus AU2700™ Chemistry-Immune Analyzer. GA is represented as % with a detectable range of 3.2. − 68.1%, and the coefficient of variation (CV) was <3.0%.

### 2.4. Statistical Analysis

Analysis of variance (ANOVA) and linear trend tests were used to compare the differences between the groups and subgroups. Spearman's correlation analysis was used to observe the relationship between GA and other variables (drinking, smoking, etc.). Linear regression analysis was used to determine the effect these variables had on GA levels, with GA as the dependent variable and drinking status (nondrinking represented as 1, drinking-occasionally as 2, and drinking-frequently as 3), smoking status (no-smoking represented as 1, smoking-occasionally as 2, and smoking-frequently as 3), age, body fat percentage, fasting plasma glucose (FPG), 2h postprandial glucose (2hPG), albumin, glutamic-pyruvic transaminase (ALT), serum uric acid (sUA), FIB-4, and triglycerides (TG) as independent variables. All *p* values were double-tailed, and *p* < 0.05 was considered as statistically significant. SPSS 21.0 software (SPSS Inc., Chicago, IL, USA) was used for all statistical analyses.

### 2.5. Feature Importance Analysis

Since linear regression cannot represent the nonlinear relationships between the GA and the independent variables, we used a decision tree regression (DTR) algorithm, a feature engineering method, to find the influence of these features/variables on GA levels. The following features such as drinking status, smoking status, age, body fat, 2hPG, albumin, ALT, sUA, and triglycerides were used. The FPG was not used because it has a significant correlation with 2hPG (*r* = 0.51, *p* < 0.001). The DTR model selects the tree's node (i.e., the feature) based on the decision criterion of mean squared error (between the true GAs and the GAs predicted by the model with the selected features). The importance of a feature refers to the (normalized) total reduction of the criterion brought about by that feature.

## 3. Results

A total of 2,314 male subjects were surveyed, of these 986 (42.6%) drank frequently and 404 (17.5%) did not drink; 1710 (73.9%) smoked frequently and 390 (16.9%) did not smoke; 169 (7.3%) neither drank nor smoked; and 825 (35.7%) both drank and smoked frequently.

The clinical data showed that systolic blood pressure (SBP), gamma-glutamyl transferase (*γ*-GT), total bilirubin test (TBIL), FIB-4, albumin, and TG levels were all significantly increased in the drinking-frequently group compared to the nondrinking group, while the 2-hour insulin (2hINS) levels were significantly lower (*p* < 0.05) (Table 1). There was a linear trend in SBP, albumin, TG, and 2hINS in these three groups. There was no significant difference in the levels of diastolic blood pressure (DBP), BMI, body fat, FPG, 2hPG, and fasting insulin among the three groups (*p* > 0.05).

The average serum GA levels for the whole study population were 13.2 ± 2.4%. The serum GA concentrations in the drinking-frequently group were significantly lower than those in the no-drinking group (13.0 ± 1.7 vs. 14.1 ± 3.7, *p* < 0.05). The subjects were further divided into subgroups based on their glucose tolerance status, age, smoking status, and body fat. GA levels of each subgroup of the drinking-frequently group, except the smoking-occasionally group, were significantly lower than those in the nondrinking group (*p* < 0.05). The results of the ANOVA analysis showed that there was a statistically significant difference in serum GA concentrations among the subgroups with different drinking statuses and that the serum GA concentrations in the no-drinking group, the drinking-occasional group, and the drinking-frequently group had a decreasing trend (Table 2). Subjects who smoked frequently had no significant difference in serum GA levels compared with those who were nonsmokers (13.2 ± 2.4 vs. 13.4 ± 2.5, *p*=0.453, not listed in the table). Analysis of smoking status showed that there was also no linear trend in serum GA levels (*p*=0.180, not listed in the table).

Spearman's correlation analysis revealed that GA levels were positively correlated with FPG, 2hPG, FIB-4, and age and negatively correlated with GA and BMI, body fat, *γ*-GT, albumin, smoking status, and drinking status, all of which were statistically significant. The correlation coefficients between GA and smoking status and drinking status were −0.040 (*p*=0.056) and −0.152 (*p* < 0.001), respectively. After adjusting for age, FPG, 2hPG, BMI, body fat percentage, *γ*-GT, TBIL, albumin, and TG, the partial correlation coefficient between GA and drinking status was −0.149 (*p* < 0.001), and −0.049 between GA and smoking status (*p*=0.020) (Table 3).

Linear regression analysis was performed using GA levels as the dependent variable and the age, body fat, FPG, 2hPG, albumin, ALT, TG, sUA, FIB-4, smoking status, and drinking status as independent variables. The method used in the regression model is “enter.” The results demonstrated that drinking status was a predictor of GA in all three models with the increments of independent variables, whereas smoking status was not a predictor of GA. The standardized regression coefficients of drinking status were −0.134 in model 1 (*p* < 0.001), −0.167 in model 2 (*p* < 0.001), and −0.144 in model 3 (*p* < 0.001) with increasing independent variables. The linear regression models also showed that age, FPG, 2hPG, body fat, albumin, FIB-4, and TGs were all independent predictors of GA levels (Table 4).

Decision tree regression analysis was conducted to determine the effects of these features on GA levels. The results of this feature importance analysis using the decision tree algorithm are shown in Table 5. The effect of drinking on GA levels (0.0283) is five times higher than that of smoking on GA (0.0057). According to the importance of features analysis, the influence of drinking is much higher than that of smoking on GA levels.

## 4. Discussion

Like HbA1c, GA is a nonenzymatic glycation product; however, it can reflect the average blood glucose level of the previous 2–4 weeks because albumin has a shorter half-life. Therefore, it is a more suitable marker of the average blood sugar levels of anemia patients since GA is not affected by red blood cells or hemoglobin [15]. Moreover, GA is also more valuable in the assessment of neonatal diabetes glycemic control [16]. However, GA levels are not exclusively affected by the average blood glucose concentrations. A series of studies have found that GA levels are associated with factors such as age, body mass index, body fat, abdominal fat, and smoking status [11, 17, 18]. A previous study showed a link between GA concentration and smoking status but did not investigate the effects of drinking status [11]. Another study showed that increased alcohol consumption was associated with elevated blood glucose levels and decreased glycosylated hemoglobin and glycated hemoglobin concentrations in 300 nondiabetic men. Smoking and drinking are very common behaviors among Chinese men, and many Chinese smokers drink alcohol frequently. In order to avoid the effects of gender, our project has fully studied the effects of alcohol and smoking on GA levels in the male population of the general community, yielding some different results.

Jinuo ethnic residents in Yunnan, China, have been found to have a high prevalence of smoking/drinking [19]. Our study analyzed the association between GA levels and drinking as well as smoking in this regional population, where a high percentage of the population frequently drink and smoke. Our survey results showed that our study population had high frequency of smoking and drinking; 42.6% of the subjects drank frequently, 73.9% smoked frequently, and 35.7% both drank and smoked frequently. Pathophysiological data has indicated that alcohol can inhibit gluconeogenesis [20]. We have demonstrated that, compared with nondrinkers, those who drank frequently had similar FPG concentrations and significantly higher 2hPG and lower levels of GA. Meanwhile, the levels of GA showed a decreasing trend in individuals who were not drinking, drinking occasionally, and drinking frequently. Therefore, serum GA concentrations in those who drink frequently cannot be explained by blood glucose levels.

The subjects were further divided into subgroups based on glucose tolerance, age, smoking status, and body fat percentage. ANOVA analysis by subgroup showed that the GA levels were also significantly lower in subjects who were drinking frequently compared to those who were not drinking. These results further illustrate that the effect of alcohol consumption on serum GA levels is independent of blood glucose, age, body fat content, and smoking status. In contrast with the findings by Koga et al. [11], our results found that men who were not smoking showed no significant difference in GA levels compared with those who smoked frequently. This may be because our study had a larger sample size.

In addition to smoking and sUA, our linear regression results showed that age, FPG, 2hPG, body fat, albumin, ALT, TG, FIB-4, and drinking status were all independent factors affecting serum GA levels. Age, FPG, and 2hPG are risk factors for elevated GA, while other factors are predictors of the decreased serum GA levels. Considering that there was no statistical difference between the smoking status and the correlation coefficient of GA, smoking status was not a predictor of serum GA level in the regression model. The result from decision tree regression is consistent with the results of the aforementioned statistical analysis, demonstrating that drinking status was more important than smoking status when applied as GA predictors.

Previous work has established that albumin is associated with inflammation and nutritional status [21], that GA is negatively correlated with C-reactive protein [16], and that thyroid function also impacts GA levels [22]. Our current analysis was unable to analyze these variables as our investigation lacked this information. Based on the results of the current study, we hypothesized that alcohol leads to a decrease in serum GA concentrations because albumin synthesis and metabolism may be affected by alcohol intake. The relationship between alcohol intake and GA needs further research. Considering the effect of alcohol on the liver, we observed a significant increase in FIB-4 in the frequent drinking group. FIB-4 was also found to be a predictor of GA decline. However, we lack a mechanism that would explain how alcohol consumption leads to an increase in FIB-4 and a further decrease in GA. TBIL and *γ*-GT are two indicators of liver damage and had higher concentrations in subjects who were drinking frequently compared to those who were not drinking. The detection of GA was not affected by TBIL or *γ*-GT but was affected by alcohol. In our study, blood samples were taken at least 8 h after fasting, so the effect of alcohol on GA test results can be ignored. A study by Inada and Koga [12] observed that drinking resulted in a decrease in GA and HbA1c. However, there is no evidence to support the hypothesis that the reduction in GA and HbA1c levels caused by drinking can slow the incidence of diabetic complications. Different from previous study, in this study, more subjects were included, analyzing the relationship between GA and alcohol consumption in Chinese men at the first time.

In summary, we found that smoking status was not a significant factor contributing to a decrease in GA concentrations. GA levels were decreased in those who were drinking frequently in both the overall study population and the subgroups with DM, normal glucose tolerance, and impaired glucose regulation. GA levels of subjects who were drinking frequently had an absolute decrease of 0.628% (95% confidence interval: 0.456% to 0.800%) compared to subjects who were not drinking. This decrease was independent of blood glucose and body fat content and should be noted when making a clinical interpretation. Classifying patients by drinking statuses such as no-drinking, drinking-occasionally, and drinking-frequently is convenient to apply, and it could effectively account for the effect of drinking on GA levels in clinical practice.

It is well known that both a high-fat diet and alcohol can induce hepatic steatosis, inflammation, and fibrosis and can also change gut microbiota [23]. Furthermore, some complicated factors may have influence on this, including the changes in the gut microbiota or alcohol induced liver disease. Many studies have shown that abnormalities in the composition of the gut microbiota might contribute to the development of type 2 DM [24]. Additionally, the gut microbiota plays an important role in both nonalcoholic fatty liver disease and alcohol-related liver disease [25]. Alcohol may affect glucose metabolism in the liver by downregulating gluconeogenesis or changing hepatic lipids, inflammatory response, and oxidative stress by inducing steatosis [26, 27]. It may also affect gut microbiota diversity and interfere with the protective effect of beneficial bacteria [28]. Alcohol-related liver disease can also change the composition and function of the gut microbiota, and treatment of the gut microbiota can restore intestinal homeostasis and improve alcohol-related liver disease [29]. The ability of the gut microbiota to directly regulate GA levels is currently unclear and basic research should be done on this topic.

## Figures and Tables

**Table 1:** Clinical characteristic by drinking status.

Parameter	Overall (*n* = 2314)	No-drinking (*n* = 404)	Drinking-occasionally (*n* = 924)	Drinking-frequently (*n* = 986)
DM	208 (9.0%)	27 (6.7%)	68 (7.4%)	113 (11.5%)^*∗*^
IGR	494 (21.3%)	80 (19.8%)	187 (20.2%)	227 (23.0%)
Age (years)	40 ± 14	47 ± 15	37 ± 13	41 ± 14^*∗*^
BMI (kg/m^2^)	22.6 ± 3.4	22.4 ± 3.3	22.8 ± 3.5	22.5 ± 3.3
Body fat (%)	19.04 ± 5.74	18.87 ± 6.17	19.16 ± 5.71	18.99 ± 5.59
SBP (mmHg)	118 ± 17	118 ± 19	116 ± 16	119 ± 17
DBP (mmHg)	75 ± 12	74 ± 12	74 ± 12	77 ± 12^*∗*^
FPG (mmol/L)	5.76 ± 1.25	5.90 ± 1.84	5.70 ± 1.16	5.75 ± 1.01
FINS (mU/L)	7.59 ± 9.95	7.24 ± 6.15	8.43 ± 13.27	6.95 ± 7.14
2hPG (mmol/L)	6.36 ± 3.03	6.28 ± 3.12	6.07 ± 2.64	6.67 ± 3.29^*∗*^
2hINS (mU/L)	35.27 ± 31.62	40.08 ± 36.46	36.04 ± 32.93	32.56 ± 27.74^*∗*^
HOMA-IR	2.00 ± 2.83	1.96 ± 2.29	2.19 ± 3.66	1.83 ± 2.03
TG (mmol/L)	1.87 ± 1.54	1.68 ± 1.22	1.83 ± 1.36	1.99 ± 1.79^*∗*^
TC (mmol/L)	5.34 ± 1.13	5.20 ± 1.12	5.27 ± 1.05	5.46 ± 1.19^*∗*^
HDL (mmol/L)	1.68 ± 0.59	1.56 ± 0.52	1.60 ± 0.51	1.81 ± 0.66^*∗*^
LDL (mmol/L)	3.30 ± 0.90	3.21 ± 0.92	3.29 ± 0.87	3.31 ± 0.93
ALT (U/L)	36.8 ± 32.6	32.1 ± 14.0	34.6 ± 16.4	46.6 ± 45.2^*∗*^
*γ*-GT (U/L)	76 ± 116	46 ± 63	58 ± 63	106 ± 156^*∗*^
TBIL (mmol/L)	11.2 ± 5.8	10.6 ± 4.8	11.4 ± 6.3	11.3 ± 5.7^*∗*^
Uric acid (umol/L)	396 ± 91	388 ± 96	397 ± 89	399 ± 91
FIB-4	1.46 ± 1.52	1.46 ± 0.91	1.16 ± 0.92	1.74 ± 2.04^*∗*^
Albumin (g/L)	47.6 ± 3.3	46.8 ± 3.4	48.0 ± 3.2	47.6 ± 3.2^*∗*^
GA (%)	13.2 ± 2.4	14.1 ± 3.7	13.2 ± 2.3	13.0 ± 1.7^*∗*^

^*∗*^Significantly different between the subjects who were not drinking and who were drinking frequently. 2hINS, 2h postprandial insulin; 2hPG, 2h postprandial plasma glucose; ALT, Alanine aminotransferase; BMI, body mass index; DBP, diastolic blood pressure; FIB-4, fibrosis-4 score; FINS, fasting insulin; FPG, fasting plasma glucose; GA, glycosylated albumin; HDL, high density lipoprotein; HOMA-IR, homeostatic model assessment-insulin resistance; LDL, low density lipoprotein; SBP, systolic blood pressure; TBIL, total bilirubin; TG, triglycerides; *γ*-GT, *γ*-glutamyl transferase.

**Table 2:** Effects of drinking status on glycated albumin levels (%, mean ± SD).

Groups	Population	No-drinking	Drinking-occasionally	Drinking-frequently	*p* linear
Glucose regulation status	NGR (*n* = 1612)	13.4 ± 1.3	12.9 ± 1.2	12.7 ± 1.1^*∗*^	<0.001
	IGR (*n* = 494)	13.9 ± 1.6	13.1 ± 1.2	12.9 ± 1.2^*∗*^	<0.001
	DM (*n* = 208)	22.6 ± 10.1	16.5 ± 6.8	14.4 ± 3.7^*∗*^	<0.001

FIB-4	<1.45 (*n* = 1548)	13.6 ± 3.1	12.9 ± 1.9	12.7 ± 1.6^*∗*^	<0.001
	≥1.45 (*n* = 766)	14.8 ± 4.1	13.9 ± 2.9	13.3 ± 1.8^*∗*^	<0.001

Age group	18–39 years (*n* = 1178)	13.0 ± 1.9	12.7 ± 1.2	12.5 ± 1.2^*∗*^	<0.001
	40–59 years (*n* = 872)	14.1 ± 3.3	13.9 ± 3.5	13.1 ± 1.8^*∗*^	<0.001
	≥60 years (*n* = 264)	15.7 ± 5.4	14.5 ± 2.2	14.2 ± 2.4^*∗*^	0.001

Smoking status	No-smoking (*n* = 390)	13.7 ± 1.9	13.1 ± 3.1	13.0 ± 2.0^*∗*^	<0.001
	Smoking-occasionally (*n* = 214)	15.2 ± 5.2	13.4 ± 1.6	13.2 ± 2.1	0.001
	Smoking-frequently (*n* = 1710)	14.3 ± 4.4	13.2 ± 2.2	12.9 ± 1.6^*∗*^	<0.001

Body fat	<25% (*n* = 1953)	14.1 ± 3.6	13.2 ± 2.1	13.0 ± 1.4^*∗*^	<0.001
	≥25% (*n* = 361)	14.2 ± 4.4	13.1 ± 3.2	12.7 ± 2.5^*∗*^	0.003

^*∗*^Significantly different between the subjects who were not drinking and who were drinking frequently. DM, diabetes mellitus; IGR, impaired glucose regulation; NGR, normal glucose regulation.

**Table 3:** Correlation analysis of glycated albumin and other variables.

	Simple correlation		Partial correlation	
	*r*	*p*	*r*	*p*
BMI	−0.065	0.002	−0.054	0.010
Body fat	−0.057	0.007	−0.084	<0.001
FPG	0.667	<0.001	0.484	<0.001
FINS	−0.031	0.133	−0.005	0.799
2hPG	0.477	<0.001	0.413	<0.001
2hINS	−0.025	0.238	−0.054	0.011
TG	−0.013	0.532	−0.009	0.671
TC	−0.150	<0.001	−0.081	<0.001
HDL	−0.070	0.001	−0.105	<0.001
LDL	−0.145	<0.001	−0.045	0.031
ALT	−0.073	<0.001	−0.040	0.057
*γ*-GT	−0.042	0.045	−0.067	0.001
TBIL	−0.038	0.068	−0.020	0.329
UA	−0.098	<0.001	−0.074	<0.001
FIB-4	0.100	<0.001	−0.045	<0.032
Albumin	−0.177	<0.001	−0.060	0.004
Smoking status	−0.040	0.056	−0.049	0.020
Drinking status	−0.152	<0.001	−0.149	<0.001

Partial correlation: after adjusting for age. BMI: body mass index, FIB-4: fibrosis-4 score; FPG: fasting plasma glucose, FINS: fasting insulin, 2hPG: 2-hour postprandial glucose, 2hINS: 2-hour postprandial insulin, *γ*-GT: *γ*-glut amyl transferase, TBIL: total bilirubin, TG: triglycerides.

**Table 4:** Linear regression to determine the variables associated with GA.

	Standardized coefficients	OR (95% CI)	*p*
Model 1			
Age	0.291	0.050 (0.043, 0.057)	<0.001
Body fat	−0.059	−0.025 (−0.041, −0.009)	0.003
Smoking status	0.002	0.007 (−0.122, 0.136)	0.911
Drinking status	−0.134	−0.443 (−0.577, −0.309)	<0.001
Model 2			
FPG	0.355	0.676 (0.603, 0.748)	<0.001
2hPG	0.288	0.180 (0.156, 0.204)	<0.001
Albumin	−0.157	−0.093 (−0.112, −0.074)	<0.001
Smoking status	0.006	0.015 (−0.069, 0.100)	0.724
Drinking status	−0.167	−0.434 (−0.522, −0.345)	<0.001
Model 3			
Age	0.191	0.026 (0.020, 0.031)	<0.001
Body fat	−0.121	−0.039 (−0.051, −0.028)	<0.001
FPG	0.381	0.722 (0.651, 0.793)	<0.001
2hPG	0.266	0.167 (0.143, 0.191)	<0.001
Albumin	−0.068	−0.040 (−0.061, −0.149)	<0.001
ALT	−0.074	−0.004 (−0.006, −0.002)	<0.001
TG	−0.048	−0.062 (−0.106, −0.018)	0.006
sUA	0.014	0.000 (−0.001, 0.000)	0.393
FIB-4	−0.053	−0.064 (−0.110, −0.018)	0.006
Smoking status	−0.010	−0.024 (−0.106, 0.059)	0.570
Drinking status	−0.139	−0.356 (−0.443, −0.270)	<0.001

2hPG, 2h postprandial glucose; ALT, Alanine aminotransferase; FPG, fasting plasma glucose; FIB-4: fibrosis-4 score; sUA, serum uric acid; TG, triglycerides.

**Table 5:** The importance of features on GA.

Features	The feature's importance
2h PG	0.416760
FIB-4	0.141971
Age	0.116355
sUA	0.091316
ALB	0.055419
BMI	0.055078
TG	0.054010
ALT	0.037208
Drinking status	0.026660
Smoking status	0.005224

2hPG, 2h postprandial glucose; ALT, Alanine aminotransferase; FPG, fasting plasma glucose; FIB-4: fibrosis-4 score; sUA, serum uric acid; TG, triglycerides.

## Data Availability

The data used to support the findings of this study are available from the corresponding author upon request.
